# Sustainable regional development from the perspective of economic resilience: Based on the impact of COVID-19

**DOI:** 10.1371/journal.pone.0314663

**Published:** 2025-01-10

**Authors:** Aotian Peng

**Affiliations:** School of Business, Anyang Normal University, Anyang, China; Zhejiang University, JAPAN

## Abstract

The process of regional economic development is marked by a sustained exposure to external disturbances. In today’s unpredictable and tumultuous global environment, such disturbances have become increasingly common, underlining the need to advance a region’s economic resilience and foster adaptive mechanisms to handle environmental flux. Comparing the typical provinces in eastern, central, western and northeastern regions during the COVID-19 epidemic period, it found that the economic resilience performance of Henan Province, which is a representative of the central region, has the following characteristics. Henan Province has the lowest total production resilience in the selected regions, but it is in the second position in terms of development potential resistance, while it is in the middle range in terms of resilience performance, and it has a weaker resistance performance in terms of the resilience indicator of the standard of living of the residents. Against this backdrop, this study undertakes an analysis and comparison of the economic resilience indicators of the 18 cities in Henan Province to suggest several refinements. It urges a promotion of the integrated development of the primary, secondary, and tertiary industries through increasing emphasis on the industrial sector, which will enable a transformation of the sectoral structuring. Further, digitization can help the upgrading of industries’ intelligence and function as a lever to raise their competitiveness. Innovation in enterprises is also crucial in enhancing industrial competitiveness, by bolstering the technological innovation capabilities of enterprises and simultaneously boosting the wage-based incomes of residents, resulting in an overall improvement in their standard of living. Also, the study emphasizes the establishment of a digital talent platform to construct a sound regional talent ecology system.

## I. Introduction

Amidst a world of unprecedented change and upheaval, compounded by the COVID-19 pandemic, the global economy faces immense challenges, wherein the rupture of global value chains and the disrupted globalization of labor play a significant role. For countries and regions deeply engaged in global labor, prioritizing measures that enable their local regions to recover from the economic contraction, and possibly from an economic negative growth crisis, in the shortest possible time remains a core interest. China, in particular, faces enormous pressure and challenges under such circumstances, and the localization of production, fragmentation of value chains, and formation of regional networks have become imminent. As pandemic policies shift, regions are competing for economic advantages.

Henan Province, as a central region, has been at the heart of the country during China’s previous long historical development cycle. It was also ahead of other regions in terms of economic development. However, during the decades of implementation of the market economy, Henan Province has gradually lagged behind the south-east. The level of economic development is lower than that of the south-east, higher than that of the west and the north-east, which is at an intermediate level. Although two decades ago, China also began to take Henan as a multiple national-level strategies implementation place, Henan was expected to have a better performance in regional economic development through the support of policies. However, the policy effect has not achieved the expected results. In the past few years, affected by changes in the external environment, Henan has even revealed some problems in the process of development, which are somewhat typical of regional development in China.

Therefore, this paper, which takes Henan Province as the key research object, aims to compare the economic resilience of Henan Province with that of typical provinces in eastern, central, western, and northeastern China, to determine where it stands in terms of national economic development. The study will then compare the different performances in economic resilience among the 18 cities in Henan Province, attempting to identify the different characteristics and problems of economic development in different regions, and clarify the driving forces for future economic development, providing support for the rapid recovery and high-quality development of the economy. The current world situation is in the midst of intricate environmental changes and the global economic recession makes it particularly important to study the economic sustainability development. The purpose of using economic resilience as a breakthrough point to explore the economic development status of a region is to provide an idea that can also be applied to the analysis of the economic development status of any region in future studies. Through the analysis of economic resilience, it explores the ways for the region’s economic development to return to the original growth mode or break through to a new growth path, and finds the impetus for the sustainable development of the regional economy. The introduction section of this paper provides an introduction to the background and significance of the research. The literature review examines the connotation of economic resilience and relevant literature, summarizes pertinent research findings, and highlights the distinctions between this study and previous investigations based on these findings. In the measurement and data source section defines the measurement methods for economic resilience as well as identifies sources for research data. The result analysis section analyzes respectively the resilience of total production, development potential, and living standards in the areas under study. Finally, in light of these analyses, the conclusion section concludes by summarizing key points from throughout this paper while also proposing future directions for further research.

## II. Literature review

“Economic resilience” is the term used to describe a region’s ability to adapt, adjust, and transform its economic development under certain shocks [1]. Thus, “economic resilience” is increasingly a topic of concern in both academic and policy circles. “Resilience”, which originally comes from the Latin word “resilio”, meaning “to bounce back”, is now commonly used as an academic concept in research. Its development has gone through different stages, including “engineering resilience”, “ecological resilience”, and “evolutionary resilience” [2]. “Engineering resilience” mainly refers to the ability of matter to recover under external forces [3]. “Ecological resilience” was first proposed by Holling [4] in 1973 in his work *Resilience and Stability of Ecosystems*. He defined “ecological resilience” as the ability of an ecosystem to maintain its original structure and function despite being influenced by natural or human-induced disturbances [5]. Later, the concept of “resilience” gradually found its way into the study of human social systems and turned into “evolutionary resilience” [6], which mainly reflects the ability of human social systems to maintain their own structure and adapt to environmental changes in the face of various perturbations [7]. Currently, the study of “resilience” is widely applied in many fields, including psychology, engineering, and mechanics.

As research on the concept of “resilience” becomes more profound, especially in the stage of “evolutionary resilience”, many scholars have proposed that resilience does not always mean returning to the original state, but instead it could also involve transforming into a new state that could be more ideal, better, and more adaptable to the environment [8]. This would not only reflect a system’s ability to maintain its own structure and function, but also the ability to respond to environmental changes in a situation where the environment changes dramatically. It would also enhance the system’s ability to adapt to the environment continually, learn and evolve [9]. “Resilience” is not just a simple static concept; it emphasizes the dynamic, continuous, and evolutionary processes involved.

In 2014, Boschma [10] proposed the concept of regional economic resilience, defining it as a region’s ability to resist external shocks and quickly return to its original growth path or find new development paths under external shocks. He also believed that this ability was a long-term capacity. Martin and Sunley [11] defined economic resilience as a dynamic self-adjusting ability and a reflection of an economic development trend, which they believed contained four dimensions: fragility, resistance, redirection, and recovery. Martin and Gardiner [12] also developed a dynamic economic resilience analysis framework and measurement methods, pointing out that economic resilience was not a static concept, but a multi-stage development process. Many scholars have applied the concept of “economic resilience” to specific regional economic development studies. Jiang et al. [13] argued that the regional economic system is not a smooth and gradual process. It tends to be continuously exposed to various shocks and disturbances. The core of enhancing economic resilience is to make adaptive adjustments in response to changes in the environment. Asongu et al. [14] measured the economic resilience of 150 countries worldwide under the impact of the COVID-19 pandemic using a resilience index. Yang et al. [15] analyzed the economic resilience of Hubei Province, which was most affected by the pandemic in the early stages, and found that the recovery speed of large cities was slower than that of small and medium-sized cities. Jiang et al. [13] measured the impact of the pandemic on the Chinese economy, and found that the Chinese economy maintained strong economic resilience under the impact of the pandemic. Geng [16] analyzed the impact of residential income disparity on economic resilience and concluded that narrowing residential income disparity would improve regional economic resilience. Li [17] proposed to enhance economic resilience by building a resilient capital market. Zhang [18] suggested enhancing regional economic resilience by deepening the role of the digital economy in fostering new dynamics. He [19] points out that the demographic dividend of population quality can significantly enhance the economic resilience of cities, while the impact of the demographic dividend of population quantity is not obvious. Cai et al. [20] comprehensively measure the regional economic resilience from four dimensions: risk index, stability index, circulation index, and innovation index. They conduct empirical analysis on the economic resilience of 31 provinces in China from 2008 to 2019. Huang [21] empirical analysis demonstrates that digital inclusive finance has a significantly positive impact on the development of regional economic resilience, and the level of innovation and entrepreneurship plays an intermediary role in the process. An et al. [22] propose that the impact of public health emergencies on regional economic resilience is firstly reflected in the impact on the service industry, and then transmitted to the manufacturing industry over time. The recovery of resilience also starts with the service industry.

The existing research on the resilience of regional economic development mainly focuses on comparisons at the global or national level, through which different performances of regional economies in terms of economic resilience are identified. Some studies further use empirical methods to investigate the reasons behind these differences in performance. This paper focuses on a particular region and compares this region to typical representatives of other economic development regions in China. Comparisons are then made between areas of this region differentiated by administrative divisions. By comparing the evaluation indicators of economic resilience, the differences in the economic development status of each region are analyzed, and suggestions for further economic development are given context of the reality of each region.

## III. Measurement and data sources of economic resilience

The application and exploration of the concept of “resilience” in the field of regional economics has broadened the horizons of research methods for regional economic development. In essence, measures of "economic resilience" include measures based on core variables, economic cycle fluctuations, and multivariate statistical models. Based on the purpose of this paper and the availability of data, a core variables-based measure of regional economic elasticity was selected. Its applicability is reflected in the construction of a sensitivity index to measure the resistance and resilience of a region’s economic resilience, as represented by macroeconomic indicators such as GDP, and using Covid-19 as an impact event. As an episodic event, Covid-19 can be used as a natural experiment to compare economic development before and after its occurrence. This paper focuses on two dimensions of "economic resilience": one dimension pertains to the ability of an economic system to resist external shocks, maintain its own structure and function, reflecting its aptitude to utilize and allocate prevailing resources. The other dimension relates to the capability of an economic system to respond rapidly to shocks, adapt itself accordingly to the external environment, and portrays its proficiency in recovering over a certain period of time. In this context, Martin [23] presented two simple indicators, that leverage changes in employment circumstances per region in order to measure the region’s restorative capacity during shock periods, while its resistance is represented by the ratio of changes in that region’s employment to shifts in national employment. The specific formula is as follows:

$$\beta =\frac{\Delta E_{r}/E_{r}}{\Delta E_{n}/E_{n}}$$ (1)

Where,

ΔE_r_ = the change of employment level in a certain region during a certain period;

E_r_ = the employment level at a certain point in a certain region;

And ΔE_n_, E_n_ = the national average level during the same period.

Drawing inspiration from Martin [16], this paper evaluates the economic resilience of selected regions by measuring their “resistance” and “recovery capacities” using a two-dimensional framework. At the same time, by referring to the measurement of economic resilience by Jiang et al. [13], Wei [24], and Wei [25], this paper selects of indicators by combining the specific reality of China and the availability and credibility of data. Firstly, to reflect the impact of external environment changes, such as the COVID-19 pandemic, on regional economies, the economic resilience is measured from three dimensions—GDP as an indicator of the total production value, fixed asset investment as a measure of development potential, and per capita disposable income as an indicator of the living standards of the residents. Secondly, to account for the impact during the shock period, the occurrence of the shock (2020) is taken as the base period (t-period) and the previous year (2019) as the t-1 period to measure the resistance capacity using the difference in values between the base and t-1 periods, and the recovery capacity using the 2021 values (t+1 period). Thirdly, based on the regional division of China by the National Bureau of Statistics, Guangdong is chosen to represent the eastern region, Hubei to represent the central region, Shaanxi to represent the western region, and Liaoning to represent the northeastern region. Finally, the economic resilience of Henan Province is compared with these regions to analyze the differences in economic development within 18 cities of Henan to identify the underlying factors driving these disparities.

To sum up, this paper measures the resistance and resilience of regional economic resilience according to the following formula:

$$\beta_{\mathrm{resis}}=\frac{(E_{r,t}-E_{r,t-1})/E_{r,t-1}}{(E_{n,t}-E_{n,t-1})/E_{n,t-1}}$$ (2)

Where,

β_resis_, β_recov_ = the economic resistance, recovery capacities;

E_r_ = the relevant indicators for a region.


$$\beta_{\mathrm{recov}}=\frac{E_{r,t+1}-E_{r,t}}{E_{r,t}}$$ (3)

E_n_ = the relevant indicators for the whole country;

t = the relevant indicators for 2020;

t-1 = the relevant indicators for 2019;

And t+1 = the relevant indicators for 2021.

According to the economic development strategy after entering the 21st century, China has divided 31 provinces, municipalities and autonomous regions (excluding Taiwan Province, the Hong Kong Special Administrative Region and the Macao Special Administrative Region) into four major economic regions, as shown in Table 1. Based on this, this paper finds out the typical provinces in the four economic regions as the research object. Then the research perspective focuses on the central Henan Province, and measures, analyzes and compares the economic resilience of all the regions included in it (Henan Province consists of 18 prefectures and cities). All the data in this paper are from *China Statistical Yearbook*, *Henan Statistical Yearbook*, and statistical yearbooks and bulletins of other Chinese provinces and cities over the years.

**Table 1:** China is divided into four economic regions.

Regions	Including provinces and autonomous regions
The east	Hebei, Beijing, Tianjin, Shandong, Jiangsu, Shanghai, Zhejiang, Fujian, Guangdong, Hainan
The middle	Shanxi, Henan, Anhui, Hubei, Jiangxi, Hunan
The west	Shanxi, Sichuan, Yunnan, Guizhou, Guangxi, Gansu, Qinghai, Ningxia, Xizang, Xinjiang, Neimenggu, Chongqing
The northeast	Liaonign, Jilin, Heilongjiang

## IV. Result analysis

### (I). Resilience analysis of total production

In accordance with Formula (2), the national level of resistance for total production value is 1. Among the selected provinces, Guangdong Province ranks the highest in terms of resistance (as shown in Table 2), but its value is still lower than 1. However, the economic recovery capacity of Guangdong Province in the following year was also lower than the national average, possibly because Guangdong’s total volume of imports and exports account for about 22% of the national total, and the sudden outbreak of the COVID-19 pandemic had a huge impact on global trade, especially import and export trade, which severely hit Guangdong, as the top-ranked province for import and export trade. Hubei Province had the lowest total production value resistance in 2020, reaching -1.6736, which is related to the initial outbreak of the COVID-19 in that area. This suggests that the pandemic had indeed caused a significant impact on the economy of the region. The total production value resistance of Liaoning Province is also relatively low, which may be related to its three-industry structure. In 2020, the proportion of the three industries in Liaoning, Henan, and Shaanxi provinces were 9.1:37.4:53.5, 9.7:41.6:48.7, and 8.7:43.4:47.9, respectively. Analyzing the composition of the three industries, it is evident that the proportion of the tertiary industry in Liaoning Province is relatively high, and during the pandemic, measures taken to restrict population mobility had a significant impact on it. According to data released by the National Bureau of Statistics, the year-on-year decrease of the added value index of the tertiary industry was significantly higher than that of the primary and secondary industries in 2020. Therefore, the contraction of the national economic output value of Liaoning Province, which has the largest proportion of the tertiary industry, was more apparent under the impact of the pandemic.

**Table 2:** Total production resilience indicators of representative provinces in China.

Regions	Total production in 2019 (RMB billion yuan)	Total production in 2020 (RMB billion yuan)	Total production in 2021 (RMB billion yuan)	Total production resistance in 2020	Total production recovery capacity in 2021
Nationwide	985333	1015986	1143670	1	0.1257
Guangdong	107671	110761	124370	0.9228	0.1229
Hubei	45828	43443	50013	-1.6736	0.1512
Shaanxi	25793	26182	29801	0.4847	0.1382
Liaoning	24909	25115	27584	0.2653	0.0983
Henan	54259	54997	58887	0.4373	0.0707

Data Source: China Statistical Yearbook and author’s calculations

Among the selected regions, Hubei Province, with the weakest total production value resistance, had the highest recovery capacity for total production value, indicating that Hubei Province has experienced a significant rebound in economic output after touching the bottom during the outbreak of the COVID-19. Meanwhile, Henan Province’s total production value recovery capacity in 2021 was the lowest among the selected regions, indicating that there is still room for improvement in its economic development over the two-year period. According to the aforementioned formula, the total production value resistance and recovery capacity of each city in Henan Province were calculated and shown in Table 3.

**Table 3:** Total production resilience indicators of various cities in Henan.

Regions	Total production in 2019 (RMB billion yuan)	Total production in 2020 (RMB billion yuan)	Total production in 2021 (RMB billion yuan)	Total production resistance in 2020	Ranking	Total production recovery capacity in 2021	Ranking
Zhengzhou	11590	11850	12691	0.7231	3	0.0225	2
Kaifeng	2364	2348	2557	-0.2227	13	-0.0069	13
Luoyang	5035	5082	5447	0.3005	8	0.0093	8
Pingdingshan	2373	2418	2694	0.6180	6	0.0192	5
Anyang	2229	2275	2435	0.6663	4	0.0207	4
Hebi	989	972	1065	-0.5442	17	-0.0169	17
Xinxiang	2918	2968	3233	0.5461	7	0.0170	7
Jiaozuo	2761	2025	2137	-8.5693	18	-0.2665	18
Puyang	1581	1621	1772	0.7934	2	0.0247	3
Xuchang	3396	3389	3655	-0.0597	12	-0.0019	12
Luohe	1578	1554	1721	-0.4926	16	-0.0153	16
Sanmenxia	1444	1430	1583	-0.3022	15	-0.0094	15
Nanyang	3815	3888	4342	0.6187	5	0.0192	5
Shangqiu	2911	2887	3083	-0.2718	14	-0.0085	14
Xinyang	2758	2770	3065	0.1323	11	0.0041	11
Zhoukou	3198	3222	3496	0.2344	9	0.0073	9
Zhumadian	2742	2843	3083	1.1815	1	0.0367	1
Jiyuan	687	691	762	0.2053	10	0.0064	10

Data Source: Henan Statistical Yearbook and author’s calculations

The ranking of the total production value resistance and recovery capacity of different cities in Henan Province is consistent. Zhumadian, Puyang, and Zhengzhou occupy the top three positions, while Jiaozuo, Hebi and Luohe are ranked lower. Zhumadian is a typical agricultural city that has long faced problems such as low industrial level and insufficient innovation momentum. In recent years, it has explored an economic development model based on the integration of the primary, secondary, and tertiary industries, driven by the primary industry. This model has achieved good results in its economic development process. For example, in Zhengyang County, the construction of a modern agricultural industry park that is the only one in the country based on peanuts, starting from “the largest county in the production of peanuts in China”, has achieved industrial upgrading, with the brand value and industrial output value of peanuts reaching “double hundred billion”. The “academician economy” development model based on the technical support of academicians and doctoral workstations has achieved good results. Puyang, as a traditional resource-based city, was listed as one of the third batch of resource-exhausted cities in China in 2011. Transforming its economic structure from relying on the petrochemical industry to finding new sources of growth has been a major challenge. Through continuous exploration, Puyang has made upgrading traditional industries and developing emerging industries as its main development direction. Science and technology innovation is the key to the transformation and development of industries and the development of emerging industries. By nurturing “specialized, refined, distinctive and new” small and medium-sized enterprises through science and technology innovation, the number of high-tech enterprises in Puyang increased by 43.7% in 2021, and the number of national technology-based small and medium-sized enterprises increased by 39.8%, which is 10.7 and 11.8 percentage points higher than the average growth rate in Henan Province, respectively. By enhancing innovation capabilities, Puyang has achieved comprehensive and high-quality transformation and become the only resource-exhausted city in Henan Province selected as an outstanding example of national transformation effects. On the other hand, Jiaozuo, as one of the first 12 resource-exhausted cities in China, has transformed its economic structure by shifting from “underground mineral resource exploration to the development of above-ground tourism resources”. However, the tourism industry has been greatly affected by the pandemic, and relying solely on tourism as the pillar industry has also affected the overall economic development of the city. Similarly, Hebi, as a resource-exhausted city, is still exploring and moving forward on the path of transformation. As a national food city in Henan Province, Luohe has made good progress in the construction of the “grain to food, agriculture to industry” industrial chain in recent years. However, its industrial chain and value chain are still in the early stages of development, and further efforts need to be made to build a more concentrated industry and upgrade its industrial and value chains.

### (II). Resilience analysis of development potential

Among the selected typical representative provinces, Hubei Province had the weakest development potential and resistance capacity, which was related to the huge impact of the COVID-19 outbreak in the early stages (as shown in Table 4). In terms of the distribution of fixed asset investment in Hubei Province, investment in infrastructure, manufacturing, and real estate development all experienced negative growth, with infrastructure and manufacturing investments both experiencing a decrease of -22.8% and -24.5%, respectively. This can be described as a severe blow to the economy. However, in 2021, Hubei Province had the highest recovery capacity and development potential. On the one hand, this was due to the severe impact of the epidemic in 2020, which significantly suppressed economic development, so there was more release space in 2021. On the other hand, it also demonstrated that the economic development of Hubei Province does have great potential. After reaching the lowest point due to the impact of the epidemic, it also demonstrated strong bottoming and rebounding capabilities. Its fixed asset investment in infrastructure, manufacturing, and real estate development increased by 9.9%, 18.9%, and 25.2%, respectively. In 2020, Guangdong Province had the strongest development potential and resilience, which can be attributed to its high level of economic development. The growth rate of fixed asset investment in infrastructure, manufacturing, and real estate development in Guangdong Province in 2020 was 11.6%, -5.1%, and 9.2%, respectively, suggesting that manufacturing investment was hit hardest by the pandemic, with negative growth, while infrastructure investment had the highest growth rate and made the largest contribution to the overall fixed asset investment in the region. Guangdong Province had the highest resilience in fixed asset investment recovery, except for Hubei Province. In contrast, Shaanxi Province had the weakest fixed asset investment recovery in 2021, with negative growth. On analyzing the distribution of fixed asset investment, it was found that real estate investment had the highest growth rate in 2020. Real estate development has been greatly affected by the macroeconomic environment in recent years, which may have led to poor performance in Shaanxi Province’s recovery potential in 2021. Henan Province’s performance in terms of development potential and resilience is second only to Guangdong Province, with intermediate performance in terms of recovery potential. The resilience indicators of development potential of each city are shown in Table 5.

**Table 4:** Development potential resilience indicators of representative provinces in China.

Regions	Fixed asset investment in 2019 (RMB billion yuan)	Fixed asset investment in 2020 (RMB billion yuan)	Fixed asset investment in 2021 (RMB billion yuan)	Development potential resistance in 2020	Development potential recovery capacity in 2021
Nationwide	643103	643103	643103	1	0.0490
Guangdong	44026	44026	44026	2.4828	0.0630
Hubei	35373	35373	35373	-6.4828	0.2040
Shaanxi	25259	25259	25259	1.4138	-0.0300
Liaoning	6369	6369	6369	0.8966	0.0260
Henan	50206	50206	50206	1.4754	0.0450

Data Source: China Statistical Yearbook and author’s calculations

**Table 5:** Development potential resilience indicators of various cities in Henan.

Regions	Fixed asset investment in 2019 (RMB billion yuan)	Fixed asset investment in 2020 (RMB billion yuan)	Fixed asset investment in 2021 (RMB billion yuan)	Development potential resilience resistance in 2020	Ranking	Development potential resilience recovery capacity in 2021	Ranking
Zhengzhou	8705	9018	8459	1.2422	15	-0.0620	17
Kaifeng	1956	2061	2331	1.8587	12	0.1310	3
Luoyang	5577	5906	5522	2.0387	6	-0.0650	18
Pingdingshan	1924	2032	2286	1.9329	9	0.1250	7
Anyang	1591	1693	1906	2.2119	2	0.1260	6
Hebi	1081	1149	1301	2.1829	4	0.1320	2
Xinxiang	2709	2882	3231	2.2077	3	0.1210	8
Jiaozuo	2369	2074	2247	-4.2868	17	0.0830	13
Puyang	1977	2083	2302	1.8539	13	0.1050	10
Xuchang	2935	2984	3196	0.5723	16	0.0710	15
Luohe	1447	1525	1729	1.8429	14	0.1340	1
Sanmenxia	2407	2540	2805	1.9060	11	0.1040	11
Nanyang	4195	4427	5007	1.9074	10	0.1310	3
Shangqiu	2811	2980	3228	2.0774	5	0.0830	13
Xinyang	3001	3175	3544	2.0073	7	0.1160	9
Zhoukou	2466	2606	2771	1.9642	8	0.0630	16
Zhumadian	2356	2510	2832	2.2518	1	0.1280	5
Jiyuan	699	566	622	-6.5813	18	0.1000	12

Data Source: Henan Statistical Yearbook and author’s calculations

Regarding the indicators measuring development potential and resilience in different cities throughout Henan Province, it can be observed that overall recovery indicators are substantially less significant when compared to resistance indicators. In terms of development potential and resilience, Zhumadian, Anyang, and Xinxiang stand out as the top performers. Zhumadian ranks second in the number of projects featured on the “2020 Key Construction Project List of Henan Province”, after Zhengzhou and Luoyang. Its integrated development model entails the combination of the primary, secondary, and tertiary sectors, driven by both project and platform strategies, which presents an eminent example for other cities in China. In the field of fixed asset investment, Anyang has exhibited remarkable performance, with investment in its strategic emerging industries increasing by 9.1%. This investment accounts for approximately 31.6% of the total industrial investment in Anyang, making it a key component of the city’s industrial structural optimization agenda. In Xinxiang, investment in the tertiary industry has effectively stimulated the growth of fixed asset investment, becoming the major driving force underpinning this growth, which exhibits a promising trend. Regarding the development potential and resilience, differences in value were generally minimal among the various cities throughout Henan Province, with most cities scoring between 0.13 and 0.1. However, it is noteworthy that Luoyang and Zhengzhou ranked last and scored negatively. These cities serve as the central and sub-central regions within Henan Province and have been backed by multiple national-level policies, yet their subpar performance regarding their development potential and resilience indicators may suggest the presence of significant underlying developmental issues. These cities have failed to fulfill their role of serving as key drivers for development and have even become the only two cities in Henan Province with negative scores for development potential and resilience. In Zhengzhou, the fixed asset investment growth rates across industries such as industry, transportation and warehousing, postal services, information transmission software, and information technology services, finance, real estate, leasing and commercial services, residential services, and other services, education, culture, sports, and entertainment have been negative. Notably, in the residential services, financial, and cultural, sports, and entertainment sectors, the growth rate has decreased sharply to -70.1%, -50.6%, and -22.1%, respectively, indicating that all areas of industry have suffered setbacks. In Luoyang, the fixed asset investment growth rate was negative across a wide range of sectors, including agriculture, forestry, animal husbandry, and fishery, industry, wholesale and retail, transportation, warehousing, and postal services, accommodation and catering, leasing and commercial services, scientific research, technology services, water resource and public facility management, education, health, social work, public administration, social security, and social organizations. In particular, the growth rates for scientific research and technology services, public administration, social security, and social organizations, as well as agriculture, forestry, animal husbandry, and fishery registered at -51.2%, -49.9%, -47.3%, respectively. These figures indicate that Luoyang’s investments in fundamental agriculture, technological innovation, and public welfare have been significantly restricted.

### (III). Resilience analysis of residents’ living standards

Resilience analysis of residents’ living standards is shown in Table 6. Regarding the resilience indicators for residents’ living standards, Hubei Province had the weakest performance, followed by Henan Province. Comparing the breakdown of per capita disposable income across provinces, Hubei Province had the lowest proportion of wage income, accounting for about 49%, and Henan Province ranked second with roughly 50%. These figures suggest that wage income may be an essential factor in ensuring the resilience of residents’ living standards. In contrast, Shaanxi Province had the strongest performance, followed by Guangdong Province. Although Guangdong Province had a higher proportion of wage income than Shaanxi Province, the proportion of transfer income in Shaanxi Province was around 27%, while in Guangdong Province, it was only about 6%. This indicator shows that the state’s transfer payments played a role in safeguarding residents’ living standards in the western region, particularly in Shaanxi Province. In terms of the resilience indicators for residents’ living standards, Hubei Province had the strongest performance, while Liaoning Province had the weakest, followed by Henan Province. Comparing the breakdown of per capita disposable income across Hubei and Henan provinces, the proportion of wage income, net operating income, net property income, and net transfer income in disposable income was similar. However, the absolute value of per capita disposable income in Hubei Province was higher than that in Henan Province, and this disparity may be the reason for differences in the performance of resilience indicators for residents’ living standards.

**Table 6:** Resilience indicators for residents’ living standards of representative provinces in China.

Regions	Per capita disposable income in 2019 (RMB yuan)	Per capita disposable income in 2020 (RMB yuan)	Per capita disposable income in 2021 (RMB yuan)	Resistance of residents’ living standards in 2020	Recovery capacity of residents’ living standards in 2021
Nationwide	30733	32189	35128	1	0.0913
Guangdong	39014	41029	44993	1.0896	0.0966
Hubei	28320	27881	30829	-0.3267	0.1057
Shaanxi	24666	26226	28568	1.3343	0.0893
Liaoning	31820	32738	35112	0.6088	0.0725
Henan	24342	24810	26811	0.4057	0.0807

Data Source: China Statistical Yearbook and author’s calculations

The performance of Henan Province’s cities in terms of resilience indicators for residents’ living standards is not entirely consistent, with most cities ranking similarly in both resilience indicators (as shown in Table 7). For example, Puyang ranked second in resistance and first in recovery capacity. However, some cities had a more significant difference in their rankings between the two indicators. For instance, Shangqiu ranked first in resistance but fifteenth in recovery capacity, while Nanyang ranked fourteenth in resistance but fifth in recovery capacity. This phenomenon indicates that the resilience of residents’ living standards is not a simple positive or negative correlation. Of particular note is Zhengzhou, the provincial capital of Henan Province, a national central city, a national pilot free trade zone, a national demonstration zone for independent innovation, and an economic integrated experimental zone for aviation ports. Despite receiving multiple national-level strategic supports, it ranked lower in both resistance and recovery capacity. One possible explanation for this could be due to the pains of transformation and upgrading in development. However, it also highlights the need for cities to further address issues related to residents’ disposable income during the development process, as improving residents’ living standards is the ultimate goal of social development.

**Table 7:** Resilience indicators for residents’ living standards of various cities in Henan.

Regions	Per capita disposable income in 2019 (RMB yuan)	Per capita disposable income in 2020 (RMB yuan)	Per capita disposable income in 2021 (RMB yuan)	Resistance of residents’ living standards in 2020	Ranking	Recovery capacity of residents’ living standards in 2021	Ranking
Zhengzhou	35942	37275	39511	0.7823	15	0.0600	18
Kaifeng	21795	22647	24573	0.8250	7	0.0850	7
Luoyang	27101	28096	30219	0.7747	16	0.0756	13
Pingdingshan	24020	24929	26869	0.7987	12	0.0778	10
Anyang	24647	25530	27365	0.7561	17	0.0718	16
Hebi	26105	27110	29362	0.8122	9	0.0831	8
Xinxiang	24562	25497	27457	0.8029	10	0.0769	12
Jiaozuo	27116	28127	30076	0.7863	13	0.0693	17
Puyang	21592	22584	24747	0.9692	2	0.0958	1
Xuchang	25949	26935	29028	0.8013	11	0.0777	11
Luohe	24625	25585	27994	0.8223	8	0.0942	4
Sanmenxia	23924	24864	26908	0.8289	6	0.0822	9
Nanyang	22638	23481	25489	0.7853	14	0.0855	5
Shangqiu	20175	21117	22699	0.9851	1	0.0749	15
Xinyang	20928	21861	23948	0.9403	4	0.0955	2
Zhoukou	18321	19143	20773	0.9463	3	0.0851	6
Zhumadian	19644	20520	22440	0.9403	4	0.0936	3
Jiyuan	29065	30013	32271	0.6881	18	0.0752	14

Data Source: Henan Statistical Yearbook and author’s calculations

## V. Conclusion

### (I). Conclusion

In the context of the global economic downturn, research on regional economic resilience has become increasingly important. This article selected typical provinces to represent the east, central, west, and northeast regions of China, and analyzed their regional economic resilience indicators. By comparing Henan Province with these typical provinces, the following conclusions can be drawn: Henan Province has a relatively weak performance in regional economic resilience. The specific reasons for its lagging performance are as follows: (1) Henan Province’s industrial structure exhibits a relatively high proportion of primary industry, thus necessitating the further integration and consolidation of the primary, secondary, and tertiary industries to promote holistic, sustained development. (2) Although Henan Province has made strides in implementing transformative measures, such as advancing manufacturing industry upgrades and strategic emergent industries, there remains much room for further development in terms of enhancing industrial competitiveness and promoting high-end, intelligent growth. (3) In order to remain innovative in the present-day market environment, enterprises in Henan Province must continue to enhance their capacity and cultivate a culture of creative ideation. (4) Given its status as a populous province, Henan Province must place a heightened emphasis on attracting and cultivating high-quality human capital, whilst continuously striving to improve the living conditions of local residents.

This paper focuses on Henan Province. The economic development resilience of all areas in Henan Province is compared and analyzed, and on this basis, targeted sustainable development proposals are put forward in the context of the actual development of the region. Compared with previous studies, this research on sustainable regional economic development is more focused in perspective and more specific in methodology. Firstly, compare and contrast the development of a region within the broader system of national economic development in order to find its niche, and then do a more detailed analysis of regional realities to propose measures for the sustainable development of the region’s economy based on the performance of economic resilience indicators. The research ideas in this paper can be generalized to the study of sustainable development in other regions.

### (II). Policy recommendations

In light of the findings reflected in this analysis, the following recommendations are posited to bolster the economic resilience of Henan Province: Firstly, Henan Province must prioritize structural overhauls of its economy, pivoting towards industrialization while retaining a focus on food security. This necessitates the integration of primary, secondary, and tertiary industries to ensure the attainment of sustainable, high-quality regional development. Secondly, Henan Province must concentrate on advancing digital industries to bolster industrial competitiveness, perpetually striving to enhance intelligent growth through technological innovation. With careful consideration of the positioning of economic entities within the industrial chain, Henan Province can successfully upgrade the established industrial sectors and promote the scale development of emerging industries alike. Thirdly, Henan Province must leverage the confluence of a variety of national-level strategies to stimulate energetic innovation and cultivate “specialized, refined, distinctive, and new” small and medium-sized enterprises. By promoting industrial competitiveness at the micro-level, the vitality of the wider market can be enhanced, creating additional job opportunities and bolstering the livelihoods of residents in the process. Lastly, Henan Province must proactively take inventory of its talent pool and closely examine the talent structure of the region overall. In light of these assessments, a comprehensive digital talent platform and a regional “talent map” can be developed, thereby attracting and retaining high-quality personnel to create a vibrant, innovative talent ecosystem that promotes the continued development of local industries.

### (III) Limitations and future work

By using the method of quantitative analysis, this article analyzes the toughness index of regional economy from the aspects of total production, development potential and residents’ living standard, and compares several typical areas. Through comparative analysis, it puts forward improvement suggestions for the development of backward regions, which provides new ideas for high-quality development of regional economy. However, at the same time, this study still has certain shortcomings, such as the selection of typical regions is subjective to some extent, and the selection of indicators describing economic resilience can be more diverse. If there are opportunities to expand this study in the future, the selection of comparative regions and related indicators will be further enriched, so as to obtain more comprehensive research conclusions.
